# LEDGF/p75 Mediates Chemoresistance in Leukemia in a Context Dependent Manner

**DOI:** 10.3390/cells15201866

**Published:** 2026-10-14

**Authors:** Thatcher Zinabu Akele, Cecilia Iglesias-Herrero, Siska Van Belle, Samantha Pretto, Frauke Christ, Zeger Debyser

**Affiliations:** 1 Advanced Disease Modelling, Targeted Drug Discovery and Gene Therapy (ADVANTAGE), Department of Pharmaceutical and Pharmacological Sciences, KU Leuven, 3000 Leuven, Belgium; thatcher.akele@kuleuven.be (T.Z.A.);; 2 Department of Microbiology, Immunology and Transplantation, Allergy and Clinical Immunology Research Group, KU Leuven, 3000 Leuven, Belgium

**Keywords:** *PSIP1*, chemoresistance, epigenetics, cell survival, leukemia

## Abstract

Lens epithelium-derived growth factor p75 (LEDGF/p75), also known as PSIP1, is a chromatin-associated protein involved in transcriptional regulation, stress adaptation, and leukemia maintenance. In KMT2A-rearranged leukemia (KMT2A-r), LEDGF/p75 tethers KMT2A fusion complex to chromatin and is essential for leukemogenesis. However, its contribution to therapy resistance across different leukemic subtypes remains poorly defined. Here, we investigated the role of LEDGF/p75 in therapy response across leukemia models with diverse genetic backgrounds. We show that LEDGF/p75 depletion sensitizes KMT2A-r T cell acute lymphoblastic leukemia (T-ALL) cells to cytarabine, whereas depletion of LEDGF/p75 in KMT2A-r B cell acute lymphoblastic leukemia (B-ALL) cells protects against cytarabine-induced apoptosis, highlighting context-specific functions of LEDGF/p75. Notably, in chronic myeloid leukemia (CML) cells harboring BCR-ABL fusion, loss of LEDGF/p75 increases sensitivity to vincristine and BCR-ABL inhibitors. Mechanistically, transcriptomic and protein analysis revealed that LEDGF/p75 depletion in CML cells is associated with reduced JAK2-STAT5 signaling, upregulation of apoptotic transcriptional programs, and suppression of proliferation and drug resistance gene signatures. Together, these findings demonstrate a context-dependent role of LEDGF/p75 in leukemic therapy resistance and identify LEDGF/p75 as a therapeutic vulnerability that could be exploited to enhance treatment sensitivity in selected leukemic subtypes, including BCR-ABL-positive CML.

## 1. Introduction

Over the past two decades, major advances in leukemia treatment have been achieved through the use of combination chemotherapy and the introduction of targeted therapies [1,2]. Improvements in molecular profiling have enabled more precise classification of leukemia subtypes and the identification of actionable genetic alterations, resulting in significant therapeutic progress in both acute and chronic leukemias [3]. Despite these advances, durable remission remains challenging, particularly in leukemias harboring high-risk genetic alterations. While targeted therapies have revolutionized leukemia treatment, their efficacy as monotherapy is often limited by the appearance of drug resistance and disease relapse [4]. Resistance to targeted therapies can arise through multiple mechanisms, including acquisition of mutations that impair drug binding, amplification of target genes, activation of alternative survival pathways, enhanced DNA damage repair, and modulation of the tumor microenvironment [5,6,7,8].

LEDGF/p75, encoded by the *PSIP1* gene, is a ubiquitously expressed protein that acts as a chromatin reader, tethering proteins to active chromatin regions [9]. *PSIP1* encodes for two main isoforms, p52 and p75 [9], both containing a PWWP domain that reads di- and tri-methylation at the 36th lysine residue of Histone 3 (H3K36me2/3). The p75 isoform presents an additional C-terminal integrase-binding domain (IBD) that functions as a protein-protein interaction hub [10,11]. Initially identified as a transcriptional coactivator involved in stress responses [12], LEDGF/p75 has since been implicated in transcriptional regulation, DNA repair, and cell survival [12,13]. It was first extensively studied as an essential host factor for HIV integration [14,15,16], and it has more recently emerged also as an oncogenic factor in multiple malignancies [17,18,19,20].

Lysine Methyltransferase 2A (KMT2A/MLL), one of the proteins that interact with LEDGF/p75, is a histone methyltransferase frequently involved in chromosomal rearrangements in hematological malignancies, generating oncogenic fusion proteins that drive aberrant transcriptional programs involving genes such as *HOXA* and *MEIS1* [21,22]. Approximately 10% of leukemia patients worldwide harbor *KMT2A* chromosomal rearrangement (KMT2A-r) [23]. LEDGF/p75 interacts with both KMT2A and Menin via its IBD domain, with Menin serving as a critical molecular adaptor that stabilizes the KMT2A-LEDGF/p75 interaction [24]. We and others have demonstrated that LEDGF/p75 is essential for the initiation and maintenance of KMT2A rearranged (KMT2A-r) leukemia, while being dispensable for normal hematopoiesis [24,25]. More recently, a divergent role for LEDGF has been described in T cell acute lymphoblastic leukemia (T-ALL), where it acts as a tumor suppressor during leukemogenesis while serving as a dependency factor for the survival of the established leukemic cells [26].

Recently, we demonstrated that depletion of LEDGF/p75 in Thp-1 cells, a KMT2A-r acute myeloid leukemia (AML) model, increases sensitivity to cytarabine and promotes apoptosis [27]. Given its role in KMT2A-r leukemia and its involvement in other malignancies [18,19,20], LEDGF/p75 presents a compelling therapeutic target. However, the contribution of LEDGF/p75 to cell survival and therapy resistance across leukemia subtypes remains poorly understood.

In this study, we investigated the role of LEDGF/p75 in therapy resistance across leukemia models with distinct genetic backgrounds. We demonstrated that depletion of LEDGF/p75 sensitizes KMT2A-r T-ALL and BCR-ABL-positive chronic myeloid leukemia (CML) cells to therapy, whereas B-cell acute lymphoblastic leukemia (B-ALL) cells displayed partial protection against cytarabine-induced apoptosis following LEDGF/p75 depletion. Mechanistically, loss of LEDGF/p75 in CML models suppresses JAK2–STAT5 signalling and reduces pro-survival transcriptional programs. Collectively, our findings identify LEDGF/p75 as a subtype-specific regulator of therapy response in leukemia and highlight its potential as a therapeutic vulnerability in selected leukemic subtypes.

## 2. Methods

### 2.1. Cell Culture

Thp-1, Kasumi-1, and K-562 cell lines were generously provided by Prof. Schwaller (Basel University, Basel, Switzerland). Nomo-1 and MV4-11 cell lines were kindly provided by Prof. Cools (KU Leuven, Leuven, Belgium), while the Nalm-6 cell line was a generous gift from Prof. Adachi (Yokohama City University, Yokohama, Japan). JURL-MK1 and U-937 cell lines were purchased from Deutsche Sammlung von Mikroorganismen und Zellkulturen (DSMZ, Braunschweig, Germany). The Karpas-45 cell line was purchased from the European Collection of Authenticated Cell Cultures (ECACC, Salisbury, UK). The SUPT-1 cell line was acquired from the National Institutes of Health reagent program (NIH; Bethesda, MD, USA), and the Jurkat cell line was obtained from the cell collection of the Center for Genomic Regulation (CRG; Barcelona, Spain). All cell lines were cultured in RPMI-1640 medium (GIBCO, Waltham, MA, USA), supplemented with 10–20% fetal bovine serum (FBS; GIBCO, Waltham, MA, USA) and 0.05 mg/mL gentamicin (GIBCO, Waltham, MA, USA). Cells were maintained at 37 °C in a humidified atmosphere with 5% CO_2_. Routine testing for mycoplasma contamination was performed using the PlasmoTest Mycoplasma Detection kit (InvivoGen, San Diego, CA, USA).

All experimental procedures involving cell lines were conducted with approval from the KU Leuven ethical committee (S699957). Cytarabine was purchased from Sigma-Aldrich (St. Louis, MO, USA), and Vincristine, Imatinib, Ponatinib, and Pimozide from MedChemExpress (Monmouth Junction, NJ, USA).

### 2.2. Viral Vector Transduction and Generation of Stable Cell Lines

Lentiviral vectors expressing shRNA targeting LEDGF/p75 were generated based on miR30. As a control, a miR30-based shRNA targeting eGFP was used. Hairpins were cloned into the PGAE-SFFV plasmid backbone with a zeocin resistance cassette. For rescuing LEDGF/p75 by back complementation (LEDGF/p75 BC), the genetic code for wild-type LEDGF/p75 that is resistant to LEDGF/p75 targeting shRNA was cloned into the PGAE-SFFV plasmid backbone with a blasticidin resistance cassette. All lentiviral vectors were produced as previously described [28]. In short, before transfection, 6.5–7 × 10^6^ of HEK293T (ATCC, Manassas, VA, USA) cells were seeded in cell culture dishes. Then 10 µM linear polyethylenimine (PEI; Polysciences, Warrington, PA, USA) was used to co-transfect 60–70% confluent HEK-293T cells with 20 µg transfer plasmid, 5 µg VSV-G envelope plasmid, and 10 µg SIV3+ packaging plasmid. After 24 h post-transfection, the medium was replaced and cells were washed with Phosphate Buffered Saline (PBS) to remove the excess plasmid. Supernatant was collected after 48 and 72 h, filtered through a 0.45 μm pore-size filter (Merck Life Science, Darmstadt, Germany), and concentrated using an Amicon Ultra-15 centrifugal filter unit, 50 kDa (Merck Life Science, Darmstadt, Germany). To generate a stable LEDGF/p75 knockdown (KD) and control cell lines, cells were transduced with concentrated lentiviral supernatant in a 1:1 volume ratio with subsequent selection with 1 µg/mL zeocin (InvivoGen, San Diego, CA, USA). To back-complement wild-type LEDGF/p75, LEDGF/p75 KD cells were transduced with the LEDGF/p75 BC vector and selected with 5 µg/mL blasticidin (InvivoGen, San Diego, CA, USA).

### 2.3. Western Blotting

Cell pellets were lysed using 1% (*w*/*v*) SDS (Sigma-Aldrich, St. Louis, MO, USA) supplemented with protease and phosphatase inhibitors (Thermo Fisher Scientific, Waltham, MA, USA), and protein concentrations were determined by BCA protein assay (Thermo Fisher Scientific, Waltham, MA, USA). Equal amounts of lysates were run on 4–20% SDS-PAGE TGX gels (Bio-Rad, Hercules, CA, USA) and transferred to nitrocellulose (Cytiva, Marlborough, MA, USA) membrane using the Trans-Blot Turbo transfer system (Bio-Rad, Hercules, CA, USA). Membranes were incubated with antibodies against LEDGF/p75 and GAPDH (Abcam, Cambridge, UK); cleaved Caspase3, AKT, STAT5, pSTAT5,pSTAT4 JAK2, pJAK2, and Bcl-xL (Cell Signaling, Danvers, MA, USA); α-Tubulin and Vinculin (Sigma-Aldrich, St. Louis, MO, USA), followed by HRP-conjugated secondary antibodies (Agilent Technologies, Santa Clara, CA, USA). Blots were developed using Clarity Western ECL Blotting Substrate (Bio-Rad, Hercules, CA, USA) and imaged with ImageQuant 800 (Cytiva, Marlborough, MA, USA).

### 2.4. RT-qPCR

Total RNA was extracted from human cell lines using the Aurum Total RNA Mini kit (BioRad, Hercules, CA, USA). 500 ng of RNA was reverse transcribed (RT) into cDNA using the High-Capacity cDNA Reverse Transcription Kit (Thermo Fisher Scientific, Waltham, MA, USA). qPCR was performed with the following primer sequences: *PSIP1* (Forward primer: GAACTTGCTTCACTTCAGGTCACA; Reverse primer: TCGCCGTATTTTTTCAGTGTAGT), *GAPDH* (forward primer: GTCTCCTCTGACTTCAACAGCG; reverse primer: ACCACCCTGTTGCTGTAGCCAA). qPCR was performed with SYBR Green and analyzed with a Light Cycler 480 (Roche Diagnostics, Mannheim, Germany). The expression level was determined relative to a standard curve and normalized to a housekeeping gene (GAPDH).

### 2.5. Proliferation Assay

2–4 × 10^4^ cells were seeded in 96-well plates and incubated overnight at 37 °C. Subsequently, they were incubated with vehicle or varying concentrations of drug for 48–72 h. For each drug, 5 or more drug concentrations were tested, and each condition was tested in triplicate. Cell proliferation was determined by the WST-1 assay according to the manufacturer (Roche Diagnostics, Mannheim, Germany). The 50% inhibitory concentration (IC_50_) was calculated by nonlinear regression analysis of dose-response curves using GraphPad Prism version 10 (San Diego, CA, USA).

### 2.6. Detection of Apoptosis via Flow Cytometry

To determine the level of apoptosis, the FITC Annexin V Apoptosis Detection Kit I (BD Biosciences, San Jose, CA, USA) was employed. 5 × 10^5^ cells/mL were seeded into a six-well plate and incubated overnight at 37 °C before treatment with vehicle or drug. After 24–48 h of treatment, cells were washed with PBS and incubated with FITC Annexin V/PI for 15 min in the dark. Samples were analyzed using Attune flow cytometry (Thermo Fisher Scientific, Waltham, MA, USA). Data were analyzed using Attune Cytometric Software (Thermo Fisher Scientific, Waltham, MA, USA).

### 2.7. Colony Formation Unit (CFU) Assay

For the CFU assay, 500 K-562 cells were seeded per well in 24-well plates containing MethoCult™ H4230 (STEMCELL Technologies, Vancouver, BC, Canada), with each experimental condition plated in duplicate. After 10 days of culture, colonies were manually scored using a microscope.

### 2.8. RNA Isolation, Library Preparation, and Sequencing

Total RNA was isolated using the Aurum Total RNA Mini Kit (BioRad, Hercules, CA, USA) and cleaned with DNAse treatment according to the manufacturer’s instructions. The sequencing was outsourced to Novogene Co. Ltd. For library preparation, the Novogene in-house NGS RNA library prep kit (PT042) was used. The library was quantified using Qubit and real-time PCR and assessed for fragment size distribution with a Bioanalyzer. Pooled libraries were then sequenced on Illumina platforms to a depth of 20 million reads.

### 2.9. RNA Sequencing Data Pre-Processing, Quality Control, and Alignment

Raw sequencing reads were obtained in FASTQ format and processed using fastp for adapter trimming and quality filtering via Q20, Q30, and GC content. Reads with adapter sequences, poly-N stretches, and low quality (Q < 20) were removed. All downstream analyses were performed on the clean files. Paired-end reads were aligned to the human reference genome GRCh38 using the splice-aware alignment tool HISAT2 (version 2.2.1) with default parameters. FeatureCounts v2.0.1 was used to count the reads mapped to each annotated gene. The output count matrix was then imported into R (v4.3.3) for downstream analysis. Raw fastq files have been deposited in the NCBI GEO (GSE325214).

### 2.10. Differential Gene Expression Analysis

Genes with less than 10 reads across all samples were filtered out. Differential expression analysis was performed using DESeq2 (v1.42.0) to compare LEDGF/p75 KD to control (Mock) K-562 and JURL-MK1 cells. Variance stabilizing transformation (VST) was applied for visualization. Results were visualized using volcano plots, and DEGs were defined as those with absolute log2 fold change > 1 and adjusted *p*-value < 0.001. Gene nomenclature was adapted using org.Hs.eg.db (v3.18.0).

### 2.11. Functional and Regulatory Network Enrichment

DEGs for functional analysis were defined as described above. GO overrepresentation analysis was performed using enrichGO, and results were visualized as bubble plots. To complement overrepresentation analysis, Gene Set Enrichment Analysis (GSEA) was performed using fgsea on ranked genes using MSigDB gene sets of interest, including C2 curated gene sets for chemical and genetic perturbations (CGP) and C6 oncogenic signatures. Enrichment results were summarized as normalized enrichment score (NES) dot plots for K-562 and JURL-MK1. Gene sets with an adjusted *p*-value < 0.05 were considered significantly enriched.

### 2.12. Oncogenic Signature Analysis

For C6 oncogenic-signature analysis, full GSEA result tables from K-562 and JURL-MK1 were imported into R and harmonized to retain gene set identifier, description, set size, normalized enrichment score (NES), and adjusted *p*-value. Shared C6 signatures were identified by intersecting gene set IDs between the two cell lines, and significance was defined as an adjusted *p*-value < 0.05. Common signatures were compared across cell lines using Venn diagrams and heatmap visualizations.

### 2.13. Chemoresistance Analysis

Chemoresistance-associated pathways were defined from the MSigDB C2 CGP collection using a keyword-based selection strategy. Gene sets were retained if pathway names contained terms related to chemotherapy response or resistance (CHEMO, RESIST, DRUG, ABC, APOPTOSIS, MULTIDRUG, VINCRISTINE, DOXORUBICIN, CISPLATIN, CYTARABINE, IMATINIB, or GLEEVEC), and additional KEGG_ACUTE_MYELOID_LEUKEMIA, was explicitly included. After removing duplicate gene set pairs, the significance of the selected chemoresistance-associated pathways was evaluated and visualized as normalized enrichment score (NES) dot plots.

### 2.14. Analysis of LEDGF/p75 Expression in Public Leukemia Transcriptomes

Processed expression data from the MILE study [29], comprising 2096 samples, was downloaded from the Gene Expression Omnibus database (GSE13159, Affymetrix HG-U133 Plus 2.0). To assign each of the four *PSIP1* probe sets to each LEDGF (p75/p52) isoform, we aligned each probe sequence to *PSIP1* RefSeq transcripts and its GRCh38 locus. Two out of the four probe sets were specific for p75, targeting the p75-specific 3′UTR (205961_s_at and 209337_at). Expression values for each probe set were log2(x + 1) transformed without further normalization. Mean LEDGF/p75 expression was defined as the mean of its two specific probe sets. Each of the leukemia classes (*n* = 1816) was plotted and compared to non-leukemic bone marrow (*n* = 74) using a two-sided Wilcoxon rank-sum test with Benjamini-Hochberg correction.

## 3. Results

### 3.1. AML Cell Lines with KMT2A Rearrangement Are Highly Sensitive to LEDGF/p75 Depletion, While LEDGF/p75 Depletion Has Minimal Impact on Cytarabine Sensitivity in Non-KMT2A-Rearranged AML Cells

We previously demonstrated that LEDGF/p75 depletion increases sensitivity to the chemotherapeutic agent cytarabine in Thp-1 cells that are driven by the KMT2A-AF9 fusion [27]. Based on the established role of LEDGF/p75 in KMT2A-r-driven leukemias, we sought to investigate if this protein plays a broader role in promoting drug resistance across distinct leukemic subtypes. Specifically, we assessed the impact of LEDGF/p75 depletion on drug sensitivity, cell viability, and apoptosis in leukemia models with or without KMT2A rearrangement, including AML, T-ALL, B-ALL, and CML. This allowed us to assess whether LEDGF/p75 modulation of chemosensitivity reflects its broader role in stress-induced survival or arises from subtype-specific pathways across different leukemia subtypes.

We first confirmed our published results [27] by showing that LEDGF/p75 depletion in Thp-1 cells increases sensitivity to cytarabine. The IC_50_ in Thp-1 Mock was 2.57 ± 0.23 μM and decreased to 1.68 ± 0.15 μM in Thp-1 cells stably depleted for LEDGF/p75 (Figure 1A,B and Figure S1A). This phenotype was rescued by back complementation with LEDGF/p75 (Figure 1A). Next, we explored the role of LEDGF/p75 in two additional acute myeloid leukemia (AML) cell lines harboring a KMT2A rearrangement: MV4-11 (KMT2A-AF4) and NOMO-1 (KMT2A-AF9). We depleted LEDGF/p75 with a LEDGF targeting short hairpin RNA (shRNA) in MV4-11 and NOMO-1 cells. However, depletion of LEDGF/p75 in these cells was challenging, and we could only obtain a partial knockdown (Figure 1C and Figure S1B,C). Both cell lines were highly sensitive to LEDGF/p75 knockdown, as the partial knockdown resulted in a significant increase of apoptosis compared to the control cell line, indicating the dependence of these cell types on LEDGF/p75 for survival and proliferation (Figure 1D,E). LEDGF/p75-depleted MV4-11 and NOMO-1 cells were difficult to maintain in culture, and considering the baseline toxicity of LEDGF/p75 KD, we were not able to compare LEDGF/p75 KD and control cells for their sensitivity to cytarabine.

We next assessed the role of LEDGF/p75 in AML cell lines not harboring the KMT2A rearrangement. We transduced Kasumi-1 and U-937 cells with LV encoding shRNA mock or targeting LEDGF/p75 and obtained an efficient knockdown with a reduction in protein expression higher than 90% (Figure 1F,G and Figure S1D,E). To evaluate the impact of LEDGF/p75 on chemoresistance, we assessed the proliferation of these cell lines after cytarabine treatment using the WST-1 assay. LEDGF/p75 knockdown had no significant effect on chemoresistance in either Kasumi-1 or U-937 cells (Figure 1H,I). Next, we assessed apoptosis following cytarabine treatment in Kasumi-1 and U-937 cells depleted for LEDGF/p75. Similarly to the proliferation, no significant difference in apoptosis was observed between LEDGF/p75-depleted cells and control cells (Figure 1J,K). Overall, these data indicate that LEDGF/p75 depletion in these two AML models lacking KMT2A rearrangement does not affect cytarabine sensitivity, implying a subtype-specific effect of LEDGF/p75.

### 3.2. LEDGF/p75 Depletion Sensitizes KMT2A-r T-ALL Cells to Cytarabine but Has Minimal Effect in KMT2A Wild-Type Models

To further explore the role of LEDGF/p75, we next analyzed models of Acute Lymphoblastic Leukemia (ALL). First, we focused on T-ALL subtypes. For this purpose, we efficiently knocked down LEDGF/p75 in three T-ALL cellular models. Karpas-45 represents the KMT2A-AF4 rearrangement, while Jurkat and Sup-T1 are two KMT2A wild-type cell lines (Figure 2A and Figure S1F–H). LEDGF/p75 depletion in Karpas-45 cells significantly sensitized them to cytarabine (IC_50_: Karpas Mock = 76.32 ± 8.32 μM vs. Karpas LEDGF/p75 KD = 53.75 ± 12.46 μM), resulting in decreased proliferation in a WST-1 assay (Figure 2B). This phenotype was partially rescued upon back complementation of LEDGF/p75 (Figure 2B). Conversely, LEDGF/p75 depletion did not affect the sensitivity to cytarabine in either Jurkat or Sup-T1 cells (Figure 2C,D). We next assessed apoptosis via flow cytometry analysis of Annexin V/PI staining. Karpas-45 cells depleted for LEDGF/p75 showed an increased level of apoptosis upon cytarabine treatment and an elevated caspase-3 protein level (Figure 2E,F). In contrast, no change in apoptosis was observed in Jurkat or Sup-T1 after LEDGF/p75 depletion and cytarabine administration (Figure 2G,H). These data indicate that the role of LEDGF/p75 in cytarabine sensitivity in T-ALL is restricted to KMT2A-r cells and has minimal effect in KMT2A wild-type T-ALL models.

### 3.3. Loss of LEDGF/p75 Protects B-ALL Cells from Cytarabine-Induced Apoptosis in Cell Lines with or Without KMT2A Rearrangement

For the B-ALL subtype, we generated LEDGF/p75-depleted SEM (KMT2A-AF4) and Nalm-6 (KMT2A-wild type) cells (Figure 3A and Figure S1I,J). Unexpectedly, LEDGF/p75 depletion in SEM cells resulted in increased proliferation upon cytarabine treatment compared to the control cells in a WST-1 assay. Restoration of LEDGF/p75 expression by back complementation partially rescued this phenotype (Figure 3B). In contrast, Nalm-6 cell proliferation was not affected by LEDGF/p75 depletion (Figure 3C). We then analyzed apoptosis by Annexin/PI staining and detection of cleaved Caspase-3. Although we observed a difference in terms of proliferation, in both SEM and Nalm-6 cells, LEDGF/p75 deficiency led to lower levels of apoptosis (Figure 3D–G). These findings indicate that the role of LEDGF/p75 in therapy response is context dependent, as its depletion partially protected both B-ALL cell lines from cytarabine-induced apoptosis.

### 3.4. LEDGF/p75 Promotes Chemotherapy and Targeted Therapy Resistance in CML Cells

Lastly, we determined the role of LEDGF/p75 in CML cell viability and response to chemotherapy. Hence, we knocked down LEDGF/p75 in K-562 and JURL-MK1 cells, cellular models of CML carrying a BCR-ABL rearrangement (Figure 4A and Figure S1K,L). Depletion of LEDGF/p75 significantly reduced proliferation in K-562 cells treated with vincristine (Figure 4B). In JURL-MK1 cells, LEDGF/p75 knockdown had a modest but significant effect on proliferation (Figure 4C). Moreover, we assessed apoptosis via Annexin V/PI staining and by detecting levels of cleaved caspase-3. Both K-562 and JURL-MK1 cells depleted for LEDGF/p75 showed increased apoptosis (Figure 4D−G). To further examine whether LEDGF/p75 depletion enhances sensitivity to targeted therapy, we treated K-562 and JURL-MK1 cells with the BCR-ABL inhibitors imatinib and ponatinib. In K-562 cells, LEDGF/p75 loss significantly enhanced the anti-proliferative effects of both inhibitors (Figure 4H−I). LEDGF/p75 depletion also increased the effect of BCR-ABL inhibition on clonogenic capacity. While imatinib and ponatinib caused only a modest reduction in colony formation in control cells, both treatments markedly reduced colony formation in LEDGF/p75-depleted cells (Figure 4J). A similar trend was observed in JURL-MK1 cells, where LEDGF/p75 depletion was associated with increased sensitivity to both BCR-ABL inhibitors (Figure 4K−L).

Together, these results indicate that LEDGF/p75 supports the survival of BCR-ABL-positive cells and contributes to therapy resistance, as its depletion increases sensitivity to vincristine and BCR-ABL inhibitors.

### 3.5. LEDGF/p75 Depletion Suppresses JAK2-STAT5 Signaling and Stress Survival Programs in CML Cells

Previously, we showed that LEDGF/p75 depletion in KMT2A-r Thp-1 cells leads to reduced expression of several components of the sphingosine-1 phosphate pathway, including AKT and ERK, which are involved in cell survival and proliferation [27]. Because LEDGF/p75 depletion altered chemosensitivity in specific leukemic subtypes, we assessed AKT expression in the cell lines showing the strongest changes in chemosensitivity. Consistent with our previous observations, LEDGF/p75 depletion resulted in reduced total AKT levels in Thp-1 cells, whereas no significant changes were detected in SEM, K-562, or Karpas-45 cells (Figure S3A).

Since the impact of LEDGF/p75 depletion on chemosensitivity in acute leukemias was largely associated with KMT2A rearrangement status, we next investigated whether LEDGF/p75 supports the survival of BCR-ABL rearranged CML cells through alternative mechanisms. To address this, we performed RNA sequencing in LEDGF/p75-depleted and control K-562 and JURL-MK1 cells. Differential gene expression analysis identified 489 and 383 significantly differentially expressed genes in K-562 and JURL-MK1 cells, respectively (Figure 5A,B and Figure S3A,B). Notably, LEDGF/p75 depletion resulted in a substantially higher number of upregulated genes (326–327) compared with downregulated genes (57–162) in either cell line (Figure 5A,B and Figure S3A,B).

Gene Ontology (GO) analysis indicated that LEDGF/p75 has slightly divergent roles between the two cell lines. K-562 is enriched for immune activation, leukocyte and lymphocyte activation, cell adhesion, and hematopoietic regulation (Figure S3C), whereas JURL-MK1 is enriched for processes related to cellular organization, cell-cell adhesion, and myeloid differentiation (Figure S3D). Gene Set Enrichment Analysis (GSEA) further explained these differences. K-562 cells, positively enriched stress-related programs, including serum deprivation, proteasome inhibition (epoxomicin), viral infection, and beta-interferon treatment, alongside apoptotic and TP53-associated response programs (Figure 5C), are consistent with the LEDGF’s established role in stress-adaptation pathways [20,30,31]. In contrast, JURL-MK1 is negatively enriched for MYC targets, E2F targets, G2M checkpoint, and DNA replication/repair pathways (Figure 5D), suggesting repression of cell-cycle progression. Despite cell line-specific differences, both cell lines shared enrichment of apoptosis-related and beta-interferon-associated programs and downregulation of signatures linked to BCL2 inhibitor and cisplatin resistance (Figure 5D).

Notably, differential expression analysis revealed a significant increase in *SLFN11* expression in LEDGF/p75-depleted K-562 cells (Figure 5A and Figure S3E). *SLFN11* has been recently identified as a potential biomarker of sensitivity to DNA-damaging agents and PARP inhibitors [32]. In contrast, only a minimal increase in *SLFN11* expression was observed in JURL-MK1 LEDGF/p75-depleted cells (Figure S3F). Overall, these results indicate that LEDGF/p75 depletion induces distinct cell type-specific transcriptional changes but commonly affects pathways related to stress responses, proliferation, and apoptosis.

To identify oncogenic pathways shared between both CML models, we compared enriched oncogenic signatures and identified 21 common gene sets (Figure 5E). These included signatures related to *HOXA9*, as well as JAK-STAT and interleukin-associated pathways. *HOXA9* is a key regulator of hematopoiesis that is frequently dysregulated in leukemia [33], and its expression is maintained by the KMT2A-LEDGF/p75 complex [25]. In addition, gene sets upregulated upon *JAK2* depletion were significantly enriched, suggesting that LEDGF/p75 depletion induces a transcriptional state resembling reduced JAK2 signaling. JAK2 is an important downstream effector of BCR-ABL that promotes leukemic cell growth and survival [34,35]. Moreover, JAK2-STAT5 signaling has been linked to tyrosine kinase inhibitor resistance and disease progression [36]. Consistently, signatures associated with suppressed interleukin signaling, including IL-15 and IL-21 pathways, were also enriched (Figure 5E).

To validate these findings at the protein level, we assessed the expression and activation of key components of the JAK-STAT pathway and the anti-apoptotic protein BCL-xL. LEDGF/p75 depletion in K-562 and JURL-MK1 cells resulted in reduced STAT5 levels and a marked decrease in phosphorylated/activated STAT5 (pSTAT5) (Figure 5F and Figure S4A,B). Activated STAT4 was strongly reduced in LEDGF/p75-depleted JURL-MK1 cells, whereas this effect was less pronounced in K-562 cells (Figure 5F and Figure S4C). While phosphorylated JAK2 was consistently decreased in both CML cell lines (Figure 5F and Figure S4D,E), the anti-apoptotic factor BCL-XL (BCL2L1) showed only modest reduction following LEDGF/p75 depletion (Figure 5F and Figure S4F). Together, these data indicate that LEDGF/p75 contributes to the maintenance of JAK-STAT signaling in BCR-ABL rearranged CML cells and that its depletion reduces activation of STAT5 and STAT4 signaling pathways.

To further investigate whether reduced STAT5 activation contributes to the increased drug sensitivity of LEDGF/p75-depleted cells, we pharmacologically inhibited STAT5 activation using pimozide. Treatment with pimozide alone resulted in a similar dose-dependent reduction in proliferation in control and LEDGF/p75 knockdown K-562 and JURL-MK1 cells (Figure 6A,B). Additionally, both K-562 and JURL-MK1 control cells showed a dose-dependent loss of activating STAT5 phosphorylation upon pimozide treatment (Figure 6C,D).

We next assessed whether pharmacological inhibition of STAT5 in control cells could reproduce the enhanced sensitivity to BCR-ABL inhibitors observed following LEDGF/p75 depletion. Combined treatment with pimozide and either imatinib or ponatinib increased drug sensitivity in both K-562 and JURL-MK1 control cells compared with treatment with either BCR-ABL inhibitor alone. Importantly, STAT5 inhibition partially phenocopied the enhanced sensitivity to BCR-ABL inhibitors observed following LEDGF/p75 loss (Figure 6E–H), supporting an association between reduced STAT5 activation and the enhanced drug sensitivity associated with LEDGF/p75 depletion.

## 4. Discussion

LEDGF/p75 is a chromatin-associated protein that promotes cell survival under diverse stress conditions, including radiation, starvation, and cytotoxic drug treatment [20,37,38,39,40]. Elevated LEDGF/p75 expression has been reported in multiple cancers and is associated with increased proliferation, clonogenic growth, migration, and resistance to drug-induced apoptosis [17,41,42]. Here, we investigated the role of LEDGF/p75 in drug resistance across leukemic subtypes, extending on our previous observations in KMT2A-rearranged AML Thp-1 cells [27].

Our results demonstrate that the contribution of LEDGF/p75 to leukemic cell survival upon treatment is highly subtype dependent. LEDGF/p75 depletion sensitized KMT2A-r AML and T-ALL cells to cytarabine, whereas AML and T-ALL cells harboring wild-type KMT2A were unaffected. In contrast, LEDGF/p75 depletion in KMT2A-r B-ALL cells partially protected against cytarabine-induced cell death. These results indicate that LEDGF/p75 does not function as a universal stress survival factor across leukemias but instead exerts subtype-specific effects likely reflecting distinct transcriptional dependencies.

Consistent with this leukemia subtype-dependent role, depletion of LEDGF/p75 in two BCR-ABL positive CML models, K-562 and JURL-MK1, sensitized cells to vincristine and increased sensitivity to the BCR-ABL inhibitors imatinib and ponatinib. In K-562 cells, this increased sensitivity was also reflected in reduced clonogenic capacity following BCR-ABL inhibition, indicating that the effect extends beyond short-term proliferation. Transcriptomic profiling revealed enrichment of stress and apoptotic transcriptional programs together with suppression of proliferation-related pathways. Several drug-resistance-associated gene signatures were also reduced, suggesting a shift toward a more therapy-sensitive transcriptional state.

Pathway analysis indicated suppression of cytokine-driven JAK-STAT signaling following LEDGF/p75 depletion, supported by reduced protein phosphorylation of JAK2 and STAT5 and decreased activation of STAT4. Because JAK2-STAT5 signaling promotes CML survival and contributes to tyrosine kinase inhibitor resistance and disease progression [34,36], these results suggest that LEDGF/p75 sustains pro-survival signaling in BCR-ABL-positive CML cells. Consistent with this, pharmacological inhibition of STAT5 with pimozide enhanced sensitivity to imatinib and ponatinib and partially phenocopied the response observed following LEDGF/p75 depletion, further supporting an association between reduced STAT5 activation and increased drug sensitivity.

LEDGF/p75 has also been implicated in genome maintenance and DNA damage repair [13]. Consistent with this role, LEDGF/p75 depletion resulted in upregulation of *SLFN11* and reduced expression of DNA repair-associated gene sets. As SLFN11 promotes replication arrest and apoptosis in response to DNA damage and is a determinant of sensitivity to several anticancer therapies [43,44], loss of LEDGF/p75 may increase replication stress and enhance drug-induced cytotoxicity. Together with suppression of JAK-STAT signaling and induction of stress responses, this may contribute to the increased drug sensitivity observed in BCR-ABL-positive CML cells following LEDGF/p75 depletion.

Targeting LEDGF/p75 is emerging as a potential therapeutic strategy due to its critical role in KMT2A-r leukemia and other malignancies, including breast and prostate cancer [19,45,46]. Drug discovery efforts targeting the PWWP domain of LEDGF have recently been reported [47], supporting the feasibility of pharmacological inhibition. LEDGF/p75 inhibition could therefore represent an alternative or complementary therapeutic strategy to recently approved Menin inhibitors, used in KMT2A-r and NPM1 mutated leukemias, which remain susceptible to the development of drug resistance [48].

Our findings broaden the therapeutic relevance of LEDGF/p75 by demonstrating its role in cell survival signaling not only in KMT2A-rearranged leukemias but also in BCR-ABL-positive CML. However, the subtype-specific effects observed here indicate that therapeutic targeting of LEDGF/p75 will likely require careful patient stratification. To complement these findings, we additionally examined LEDGF/p75 expression in the publicly available MILE leukemia cohort [29]. LEDGF/p75 was expressed across the different leukemia subtypes, although expression levels varied between disease groups (Figure S5). T-ALL showed increased expression compared with non-leukemic bone marrow, whereas lower expression was observed in CML and CLL, with additional subtype-specific differences across AML and B-ALL. These observations further support the heterogeneity of LEDGF/p75 expression across leukemia subtypes and indicate that its functional contribution to therapy response may not be solely determined by its expression level.

Although this study provides important insights into the role of LEDGF/p75 in leukemic drug resistance, several limitations should be considered. Efficient depletion of LEDGF/p75 was technically challenging in some cell lines, resulting in incomplete knockdown and limiting the depth of mechanistic analyses in certain models. In addition, our conclusions are primarily based on established leukemia cell lines. This is particularly relevant for the B-ALL findings, where LEDGF/p75 depletion was associated with partial protection against cytarabine-induced cell death. As these observations were obtained from a limited number of B-ALL models, they should not be interpreted as evidence for a general tumor-suppressive role of LEDGF/p75 in B-ALL. Future studies using patient-derived samples and in vivo models will therefore be important to validate and extend these findings.

## 5. Conclusions

In conclusion, our study identifies LEDGF/p75 as a leukemia subtype-specific regulator of therapy response. Beyond its established role in KMT2A-r leukemias, LEDGF/p75 also supports survival signaling in BCR-ABL-positive CML, with reduced JAK2-STAT5 activation and altered stress-adaptation programs associated with LEDGF/p75 loss. These findings highlight LEDGF/p75 as a potential therapeutic vulnerability in selected leukemic subtypes.

## Figures and Tables

**Figure 1:**
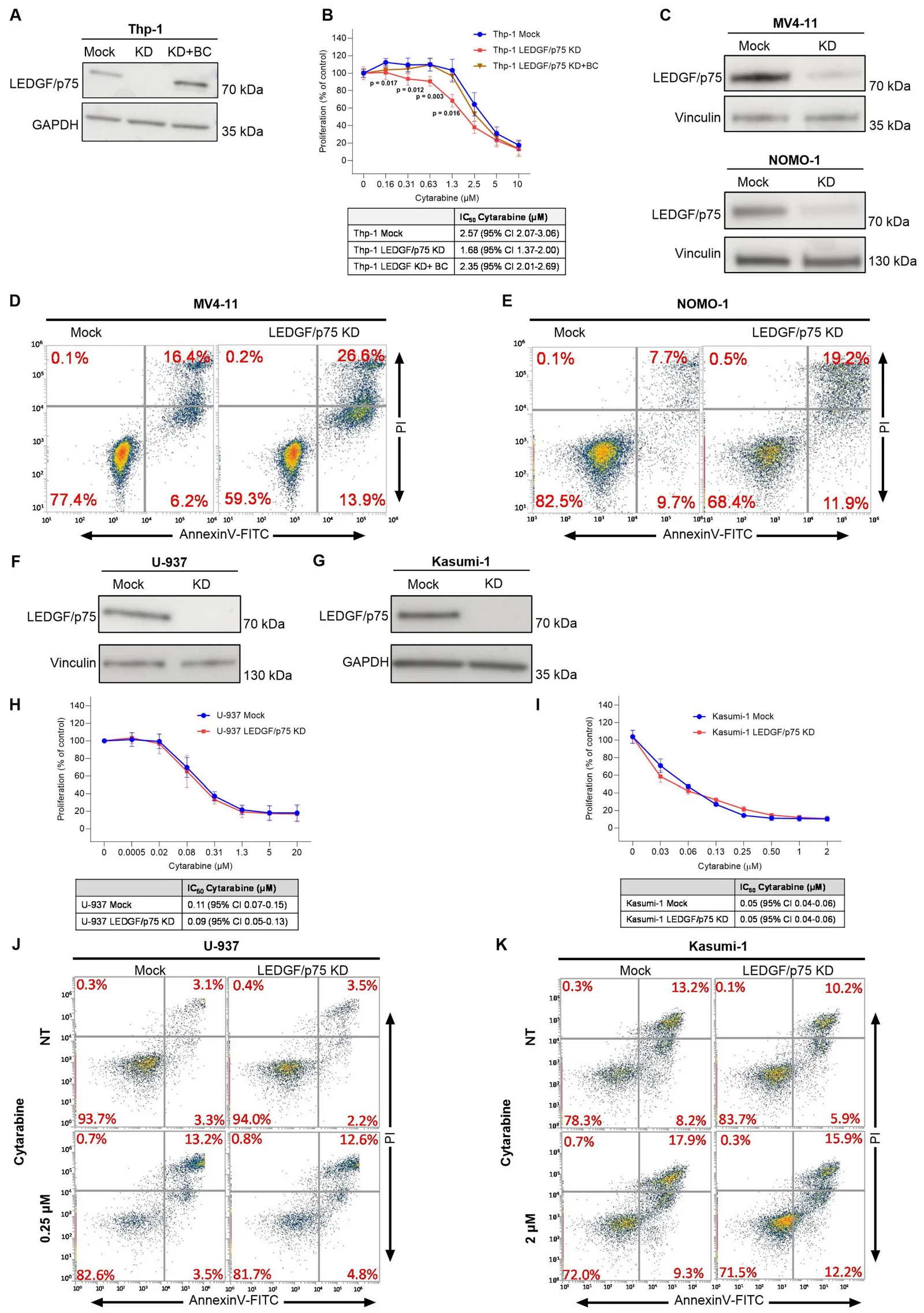
LEDGF/p75 depletion sensitizes KMT2A-r AML cells but not KMT2A WT cell lines to cytarabine. (**A**) Immunoblot showing LEDGF/p75 levels in Thp-1 cells transduced with control vector (Mock), a shRNA vector resulting in LEDGF/p75 knockdown (LEDGF/p75 KD), or Thp-1 cells depleted and rescued by back complementation (LEDGF/p75 KD + BC). GAPDH was used as loading control (**B**) WST-1 proliferation assay evaluating cell viability after 72 h of cytarabine treatment. The IC_50_ of cytarabine in each condition with 95% confidence intervals is shown in the table (below), (mean ± SD from *n* = 3 independent replicates), unpaired two-tailed Student’s *t*-test compared with Thp-1 mock data). (**C**) Immunoblot validation of LEDGF/p75 expression levels in MV4-11 and NOMO-1 cells after transduction with lentiviral vectors encoding LEDGF/p75 targeting shRNA. Vinculin was used as loading control, as indicated. (**D**,**E**) Representative flow cytometry plot of apoptosis via Annexin V and PI staining showing apoptosis in MV4-11 (**D**) and NOMO-1 (**E**) cells after transduction with lentiviral vectors encoding LEDGF/p75 targeting shRNA (*n* = 3 biological replicates). (**F**) Immunoblot validation of LEDGF/p75 depletion in U-937 cells. Vinculin was used as loading control. (**G**) Immunoblot validation of LEDGF/p75 expression levels in Kasumi-1 cells. (**H**) WST-1 proliferation assay measuring cell viability after 48 h of cytarabine treatment of U-937 cells (*n* = 3 biological replicates); IC_50_ values are shown in the table (below). (**I**) WST-1 proliferation assay of Kasumi-1 cells treated with cytarabine for 48 h. *n* = 3 biological replicates. IC_50_ values are shown in the table (below). (**J**,**K**) U-937 (**J**) and Kasumi-1 (**K**) (Mock and LEDGF/p75 KD) were treated for 24 h with 0.25 µM and 2 µM cytarabine, respectively, and stained with Annexin V-FITC/PI. The level of apoptosis was measured by flow cytometry. Percentages of cells in each quadrant correspond to necrotic (upper left), late apoptotic (upper right), early apoptotic (lower right), and live cells (lower left). *n* = 3 biological replicates.

**Figure 2:**
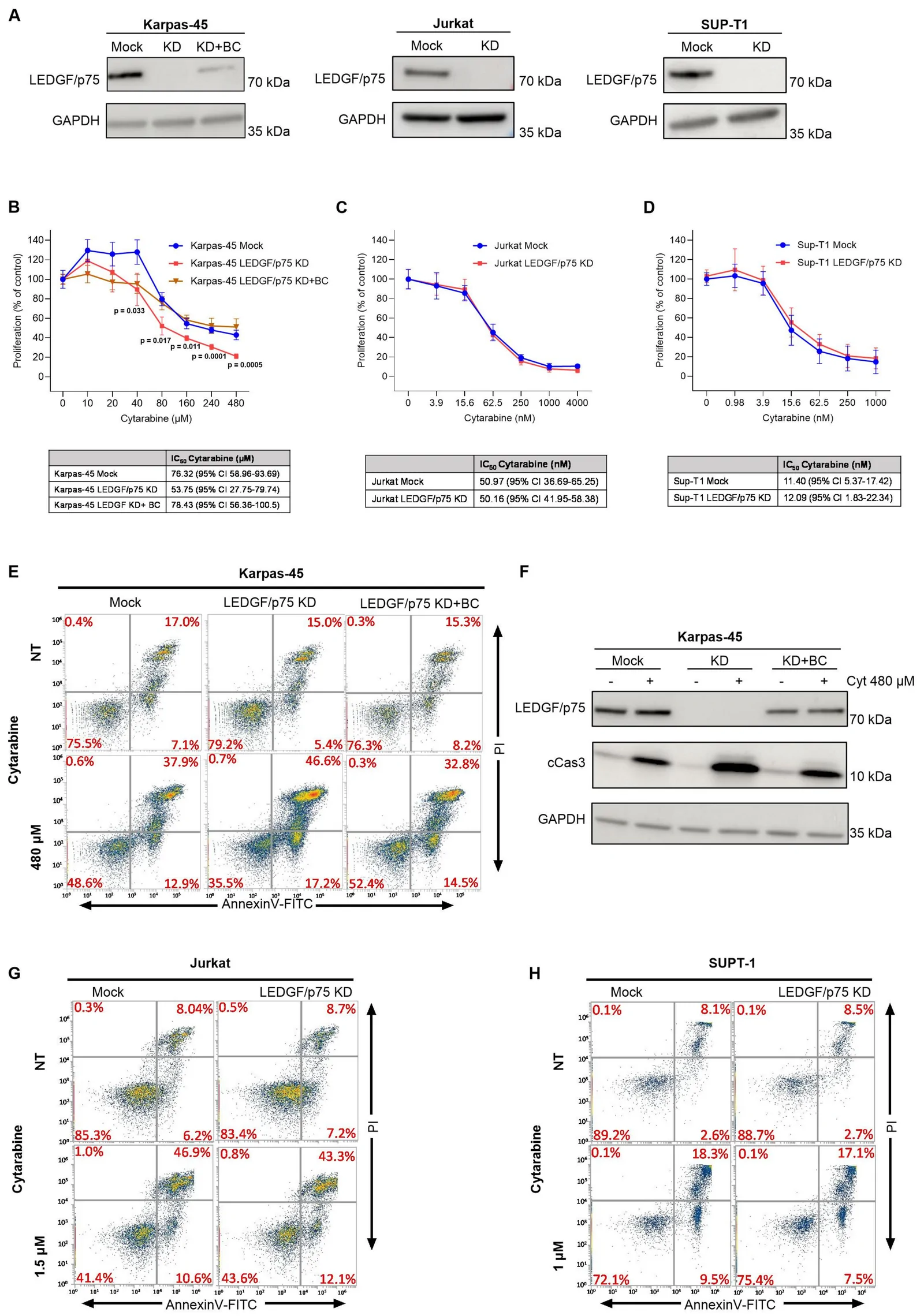
LEDGF/p75 affects chemoresistance of T-ALL KMT2A-AF4 cells. (**A**) Immunoblot of LEDGF/p75 expression levels in Karpas-45, Sup-T1, and Jurkat cells transduced with a control vector (Mock) or an shRNA vector targeting LEDGF/p75 (LEDGF/p75 KD). Back complementation (KD + BC) was performed in Karpas-45 cells to restore LEDGF/p75 expression. GAPDH was used as a loading control. (**B**–**D**) WST-1 proliferation assay showing cell viability following cytarabine treatment during 72 h for Karpas-45 cells (**B**), and 48 h for Jurkat (**C**) and Sup-T1 (**D**) cells. IC_50_ values for cytarabine with 95% confidence intervals are shown in the tables (bottom) (mean ± SD from *n* = 3 biological replicates, unpaired two-tailed Student’s *t*-test compared with Karpas-45 mock data). (**E**) Representative flow cytometry plot of apoptosis via Annexin V and PI staining in LEDGF/p75 WT, KD, and KD + BC Karpas-45 cells treated for 24 h with 480 µM of cytarabine (*n* = 3 biological replicates). (**F**) Levels of LEDGF/p75, activated caspase-3 (cCas3), and GAPDH (as loading control) measured by immunoblotting of Karpas-45 cell line extracts treated for 24 h with 480 µM of cytarabine (*n* = 3 biological replicates). (**G**,**H**) Representative flow cytometry plot of apoptosis via Annexin V and PI staining in Jurkat (**G**) and Sup-T1 (**H**) cells after 24 h of 1.5 µM or 1 µM cytarabine treatment, respectively. Percentages of cells in each quadrant correspond to necrotic (upper left), late apoptotic (upper right), early apoptotic (lower right), and live cells (lower left). *n* = 3 biological replicates.

**Figure 3:**
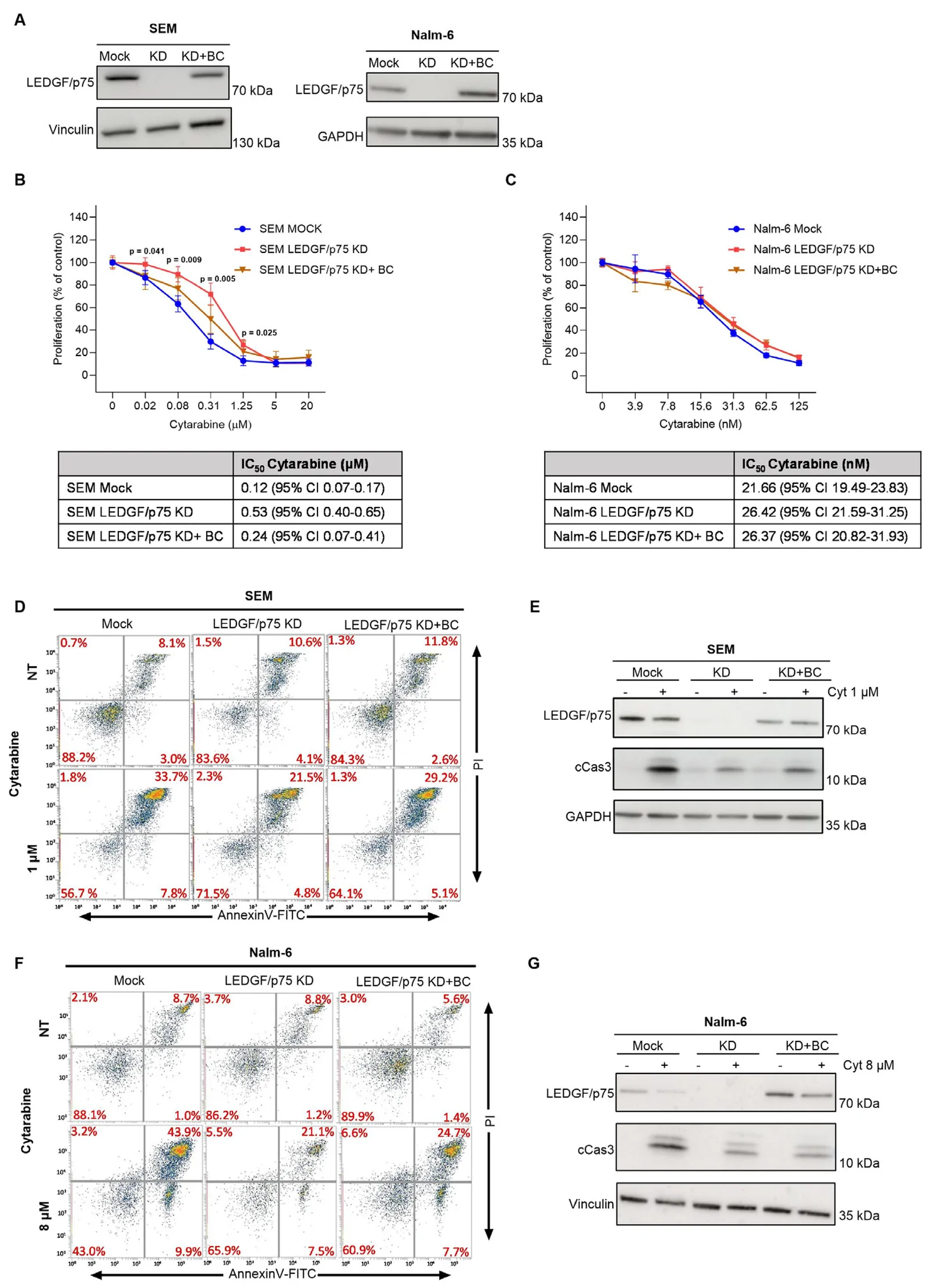
LEDGF/p75 depletion increases chemoresistance in B-ALL KMT2A-AF4 cells. (**A**) Validation of LEDGF/p75 expression levels in SEM and Nalm-6 cells following transduction with control (Mock), knockdown (KD), or back complementation (KD + BC) vectors, as determined by immunoblotting. Vinculin or GAPDH was used as a loading control. (**B**,**C**) Proliferation assay of SEM (**B**) and Nalm-6 (**C**) cells treated with cytarabine for 48 and 72 h, respectively. IC_50_ values with 95% confidence intervals are shown in the tables (*n* = 3 biological replicates, mean ± SD, unpaired two-tailed Student’s *t*-test compared with SEM mock data). (**D**–**G**) apoptosis analysis of SEM and Nalm-6 cells after 24 h of 1 µM or 8 µM cytarabine treatment, respectively (*n* = 3 independent replicates). (**D**,**F**) Representative flow cytometry plot of apoptosis via Annexin V and PI staining in SEM (**D**) and Nalm-6. Percentages of cells in each quadrant correspond to necrotic (upper left), late apoptotic (upper right), early apoptotic (lower right), and live cells (lower left) (**E**,**G**) Immunoblot analysis of LEDGF/p75 and cleaved caspase-3 (cCas3) levels in SEM (**E**) and Nalm-6 (**G**). Vinculin or GAPDH was used as a loading control. *n* = 3 biological replicates.

**Figure 4:**
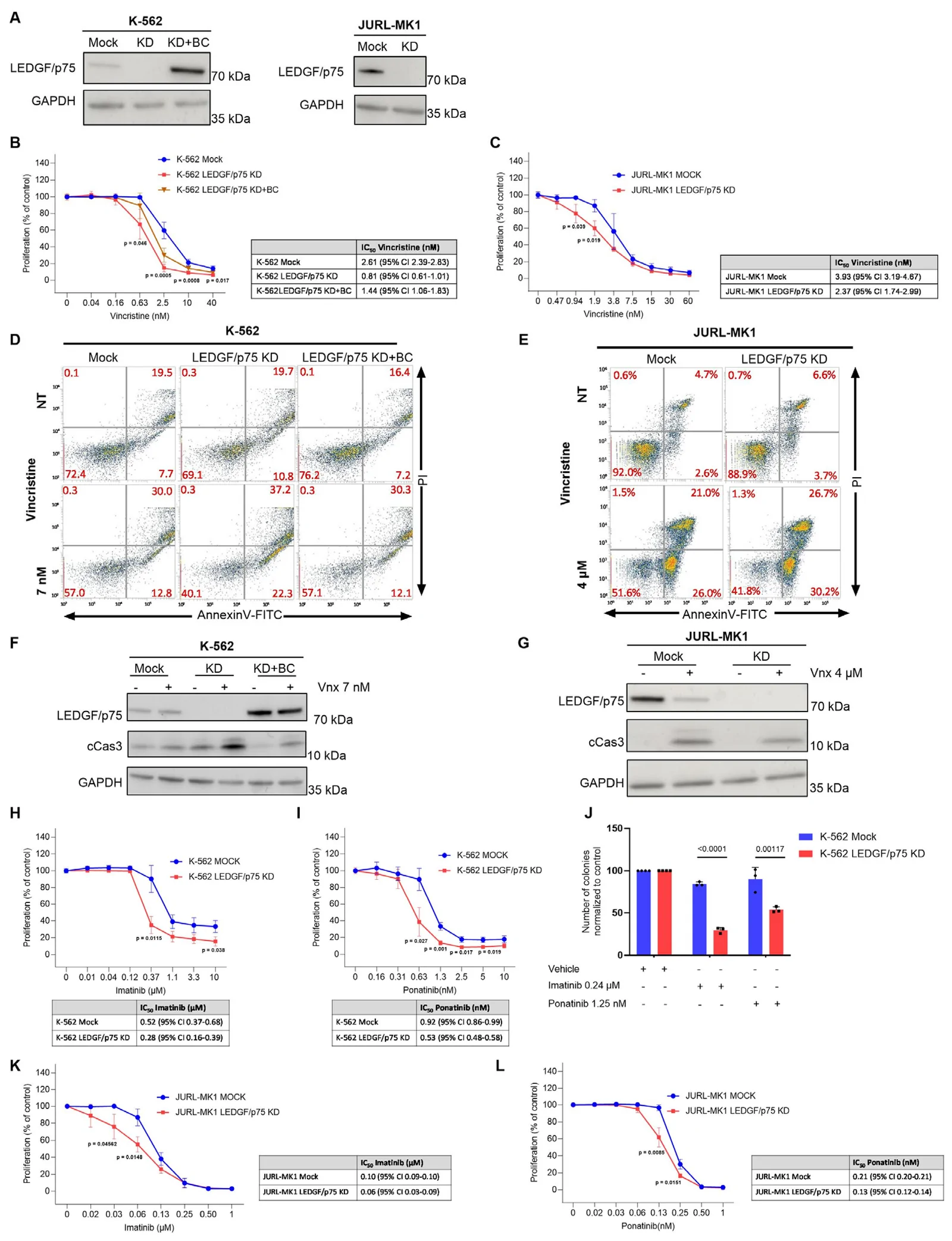
LEDGF/p75 depletion sensitizes CML cells to chemotherapy and BCR-ABL inhibitors. (**A**) Immunoblot confirming LEDGF/p75 knockdown in K-562 and JURL-MK1 cells. (**B**,**C**) WST-1 proliferation assay measuring cell viability after 72 h of vincristine treatment in K-562 (**B**) or JURL-MK1 (**C**) cells. IC_50_ values for vincristine with 95% confidence intervals are shown in the tables. Data are presented as mean ± SD from *n* = 3 biological replicates, unpaired two-tailed Student’s *t*-test compared to the mock data. (**D**,**E**) Representative flow cytometry plot of apoptosis via Annexin V and PI staining in K-562 (**D**) and JURL-MK1 (**E**) cells, after 24 h of vincristine treatment. *n* = 3 biological replicates. F-G) Expression of caspase-3 (cCas3) after 24 h of vincristine treatment in K-562 (**F**) or JURL-MK1 (**G**) cells (*n* = 3). H-I) WST-1 proliferation assays assessing the response of K-562 Mock and LEDGF/p75-depleted cells to increasing concentrations of imatinib (**H**) and ponatinib (**I**) for 72 h. Mean ± SD from *n* = 3 biological replicates, unpaired two-tailed Student’s *t*-test compared to the mock data. (**J**) Colony-forming assay of K-562 Mock and LEDGF/p75-depleted cells following treatment with vehicle, 0.24 µM imatinib, or 1.25 nM ponatinib. Colony numbers were normalized to the corresponding vehicle-treated control. *n* = 3 biological replicates were plotted. Statistical analysis was performed using an unpaired two-tailed Student’s *t*-test. (**K**,**L**) WST-1 proliferation assays assessing the response of JURL-MK1 Mock and LEDGF/p75-depleted cells to increasing concentrations of imatinib (**K**) and ponatinib (**L**) for 72 h. Corresponding IC_50_ values with 95% confidence intervals are shown in the tables. Data are presented as mean ± SD from *n* = 3 independent biological experiments, unpaired two-tailed Student’s *t*-test compared to the mock data.

**Figure 5:**
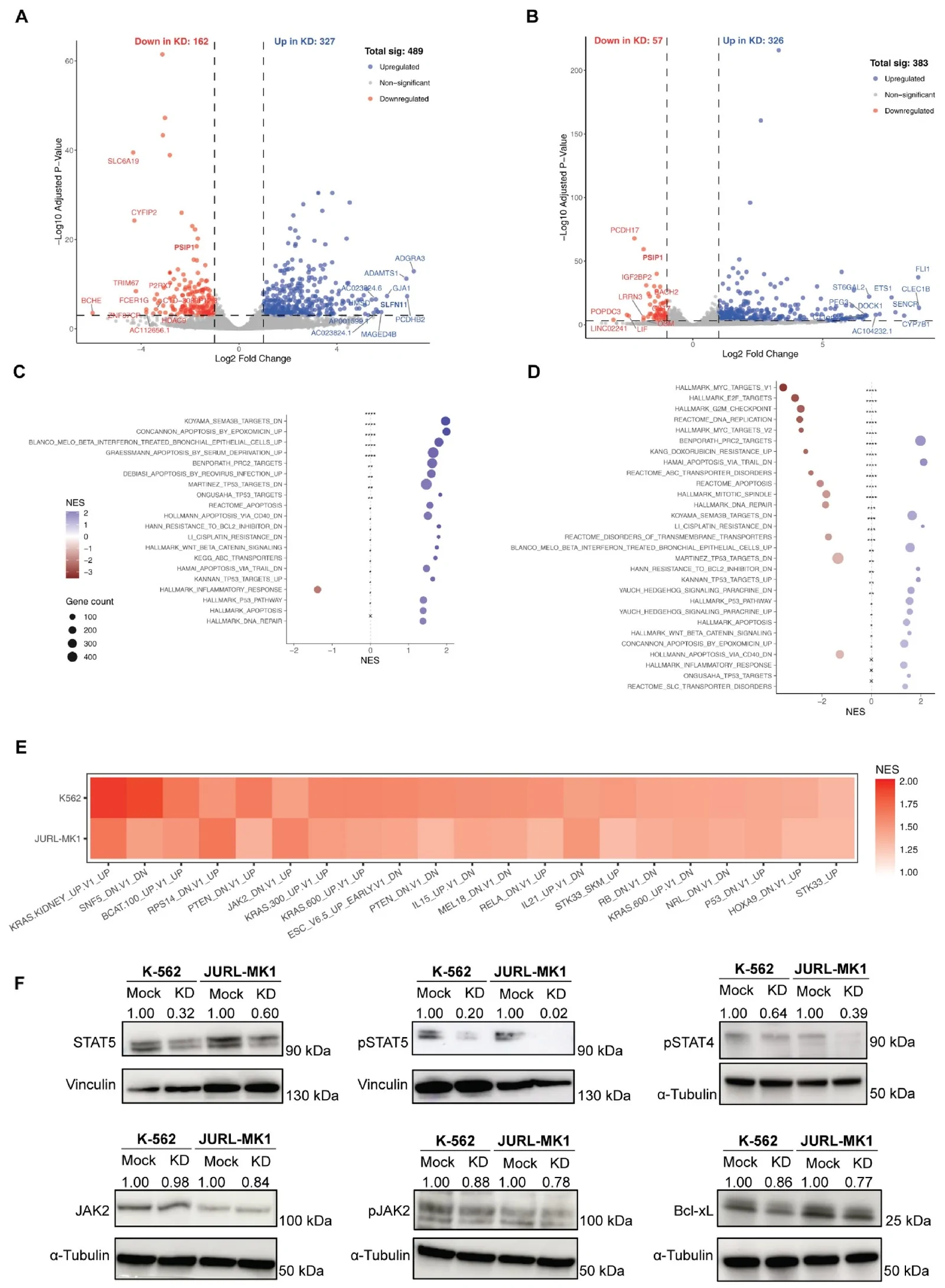
RNA-seq analysis of LEDGF/p75-depleted K-562 and JURL-MK1 cells shows altered JAK2-STAT5 signaling. (**A**,**B**) Volcano plot of differentially expressed genes between LEDGF/p75-depleted and control cells for K-562 (**A**) and JURL-MK1 (**B**) cells. Cutoff was set at Log2 fold change > 1 and adjusted *p*-value < 0.0001 (*n* = 4). Genes downregulated in KD are shown in red, upregulated genes in blue, and non-significant genes in grey. (**C**,**D**) Gene set enrichment analysis (GSEA) of chemical and genetic perturbation signature for K-562 (**C**) and JURL-MK1 (**D**). Dot size, represents gene size and color indicates NES. (**E**) Heat map showing shared enriched oncogenic signatures in K-562 and JURL-MK1 following LEDGF/p75 depletion (NES scale shown). (**F**) Representative immunoblots showing STAT5, pSTAT5, and pSTAT4, JAK2, pJAK2 and BCL-xL (BCL2L1) levels in K-562 and JURL-MK1 control (Mock) and LEDGF/p75 KD cells. Vinculin and α-tubulin were used as loading controls. Densitometric values are indicated above each lane and represent relative protein levels normalized to the corresponding loading control and expressed relative to the mock sample for each cell line (*n* = 3).

**Figure 6:**
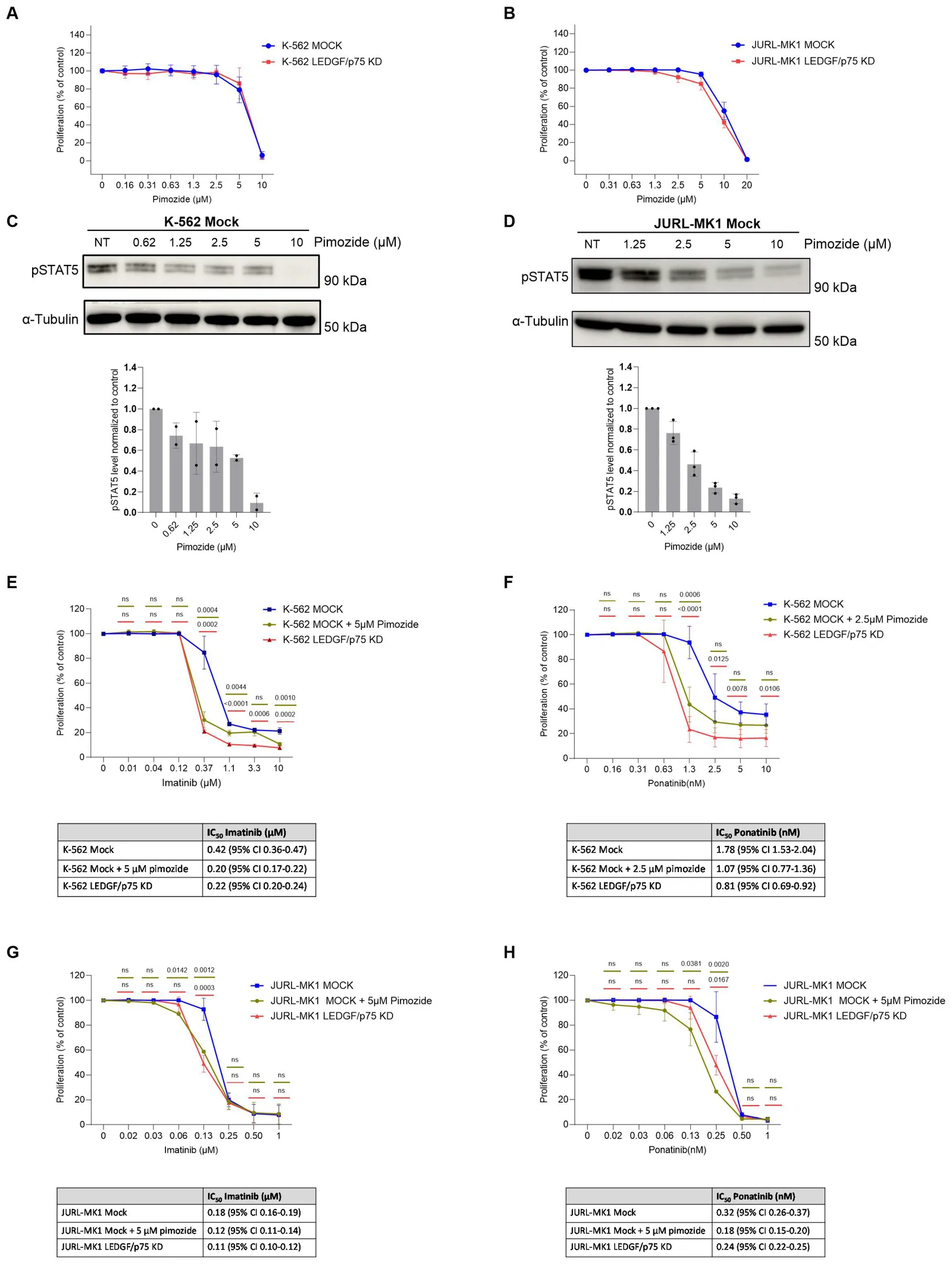
Pharmacological inhibition of STAT5 enhances the response of CML cells to BCR-ABL inhibitors. (**A**,**B**) WST-1 proliferation assays of K-562 (**A**) and JURL-MK1 (**B**) Mock and LEDGF/p75-depleted cells following 72 h treatment with increasing concentrations of the STAT5 inhibitor pimozide. (**C**,**D**) Representative immunoblots showing pSTAT5 levels in K-562 (**C**) and JURL-MK1 (**D**) Mock cells following treatment with increasing concentrations of pimozide. α-Tubulin was used as a loading control. Quantification of pSTAT5 levels normalized to α-Tubulin and to the untreated control is shown below the corresponding blots (*n* = 2 for K-562 and *n* = 3 for JURL-MK1). (**E**,**F**) Proliferation of K-562 Mock cells treated with imatinib (**E**) or ponatinib (**F**) alone or in combination with pimozide, compared with LEDGF/p75-depleted cells treated with the corresponding BCR-ABL inhibitor. K-562 cells were treated with 5 μM pimozide in combination with imatinib and 2.5 μM pimozide in combination with ponatinib (*n* = 3 for Imatinib and *n* = 4 for ponatinib is plotted). (**G**,**H**) Proliferation of JURL-MK1 Mock cells treated with imatinib (**G**) or ponatinib (**H**) alone or in combination with 5 μM pimozide, compared with LEDGF/p75-depleted cells treated with the corresponding BCR-ABL inhibitor (*n* = 3 of biological replicates). Corresponding IC_50_ values with 95% confidence intervals are shown in the tables. (**E**–**H**), statistical analysis was performed using one-way ANOVA with Dunnett’s multiple comparisons test. Exact *p*-values are indicated in the graphs; ns, not significant.

## Data Availability

The data presented in this study are openly available in [NCBI’s Gene Expression Omnibus repository] at [https://www.ncbi.nlm.nih.gov/geo/query/acc.cgi?acc=GSE325214] (accessed on 14 June 2026), reference number [GSE325214]. Additional data generated in this study is available from the corresponding author Zeger Debyser (zeger.debyser@kuleuven.be).

## Supplementary Materials

The following supporting information can be downloaded at: https://www.mdpi.com/article/10.3390/cells15201866/s1, Figure S1: RT-qPCR validation of LEDGF/p75 depletion across leukemic cell lines, Figure S2: AKT level in LEDGF/p75 depleted leukemic cell lines. Figure S3: RNA-seq profiling of LEDGF/p75 depleted CML cell lines. Figure S4: Quantification of JAK2-STAT5 pathway components following LEDGF/p75 depletion in CML cell lines. Figure S5: LEDGF/p75 expression in microarray across hematological malignancies in the MILE cohort.
