# Supplementary material for: LEDGF/p75 Mediates Chemoresistance in Leukemia in a Context Dependent Manner
Source: Cells. 2026 Oct 14;15(20):1866. doi: 10.3390/cells15201866 (PMC13703467; doi:10.3390/cells15201866)
Supplement: Supplementary file 1 [file cells-15-01866-s001.zip › cells-4454353-supplementary.pdf]

Supplementary figures

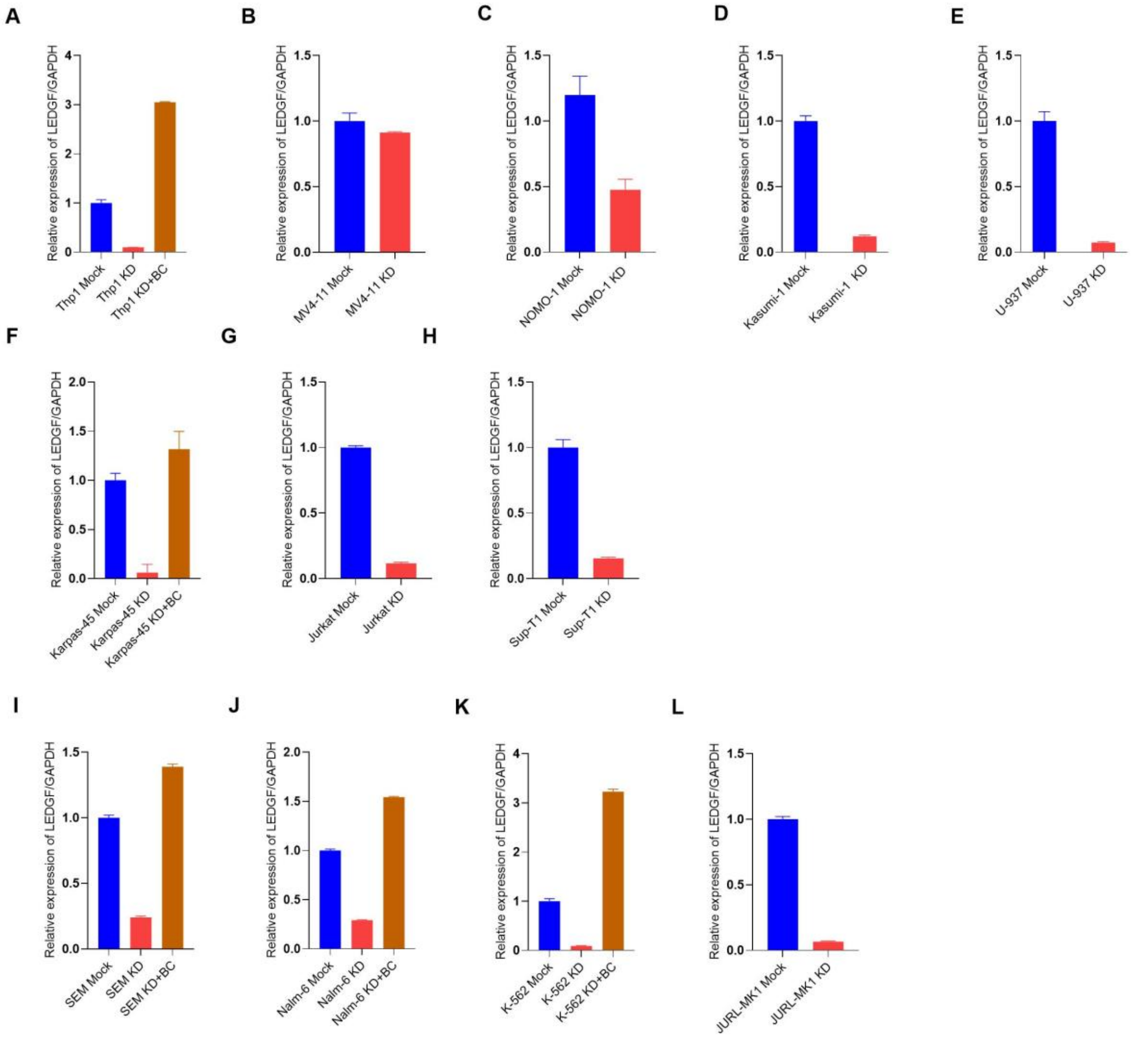

Figure S1. RT-qPCR validation of LEDGF/p75 depletion across leukemic cell lines. Relative messenger RNA (mRNA) expression was measured by RT qPCR and normalized to gapdh respectively. Bars show Mock (blue) and LEDGF/p75 KD (red) conditions in the indicated cell lines, with back complementation/rescue (KD+BC) shown where available (orange). (A) THP 1, (B) MV4-11, (C) NOMO-1, (D) Kasumi-1, (E) U-937, (F) Karpas-45, (G) Jurkat, (H) Sup-T1, (I) SEM, (J) Nalm-6, (K) K-562, (L) JURL-MK1.

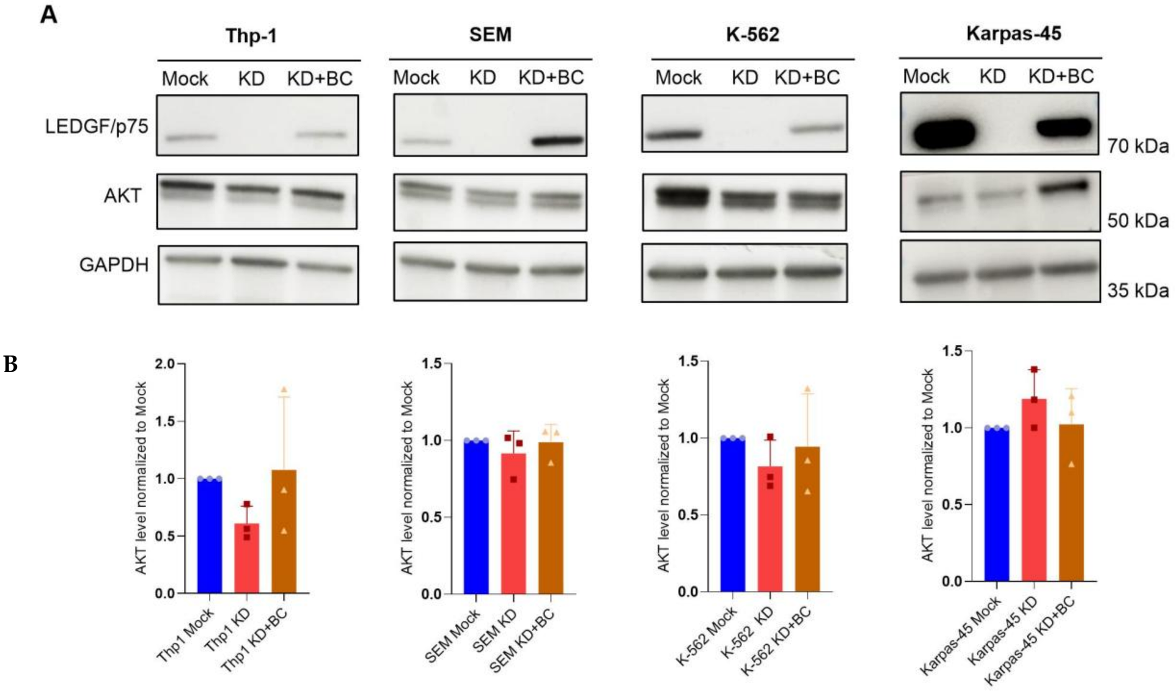

Figure S2. LEDGF/p75 depletion reduces AKT levels in Thp-1 cells but not in other leukemic cell lines. (A) Western blot analysis showing total AKT levels in Thp-1, SEM, K-562, and Karpas-45 cells transduced with a control vector (Mock), after LEDGF/p75 knockdown (KD) or back complementation (KD+BC). GAPDH was used as a loading control. (B) Quantification of AKT protein levels normalized to Mock controls. One representative blot is shown (n=3).

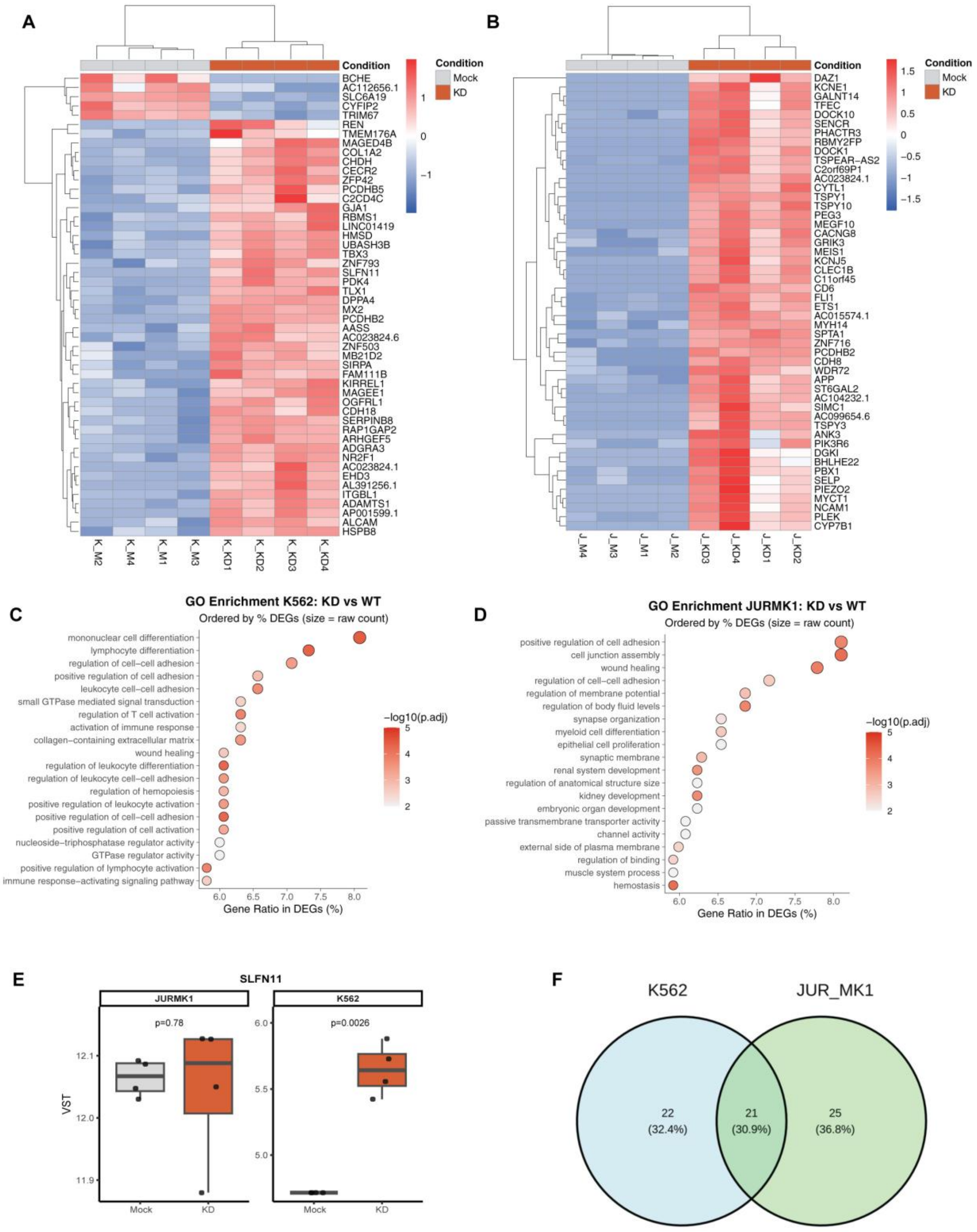

Figure S3. color represents the adjusted p-value (padj). (E) Boxplots of SLFN11 using DESeq2 variance-stabilized expression values (VST) in K562 and JURL-MK1 cells under WT and LEDGF knockdown conditions. Points represent individual replicates; boxes show median and interquartile range. Adjusted p values are indicated. (F) Venn diagram showing overlap of shared enriched C6 oncogenic signatures between K-562 and JURL-MK1 following LEDGF/p75 de pletion.RNA-seq profiling of LEDGF/p75 depleted CML cell lines. (A-B) Heat map showing the top 50 differentially expressed genes in K-562 (A) and JURL-MK1 (B) cells comparing MOCK (control) and LEDGF/p75 KD conditions (n=4).

(C-D) Gene ontology (GO) enrichment analysis of the top 20 biological processes affected by LEDGF/p75 depletion in K-562 (C) and JURL MK1 (D). The x-axis shows the percentage of gene ration in DEG and the dot.

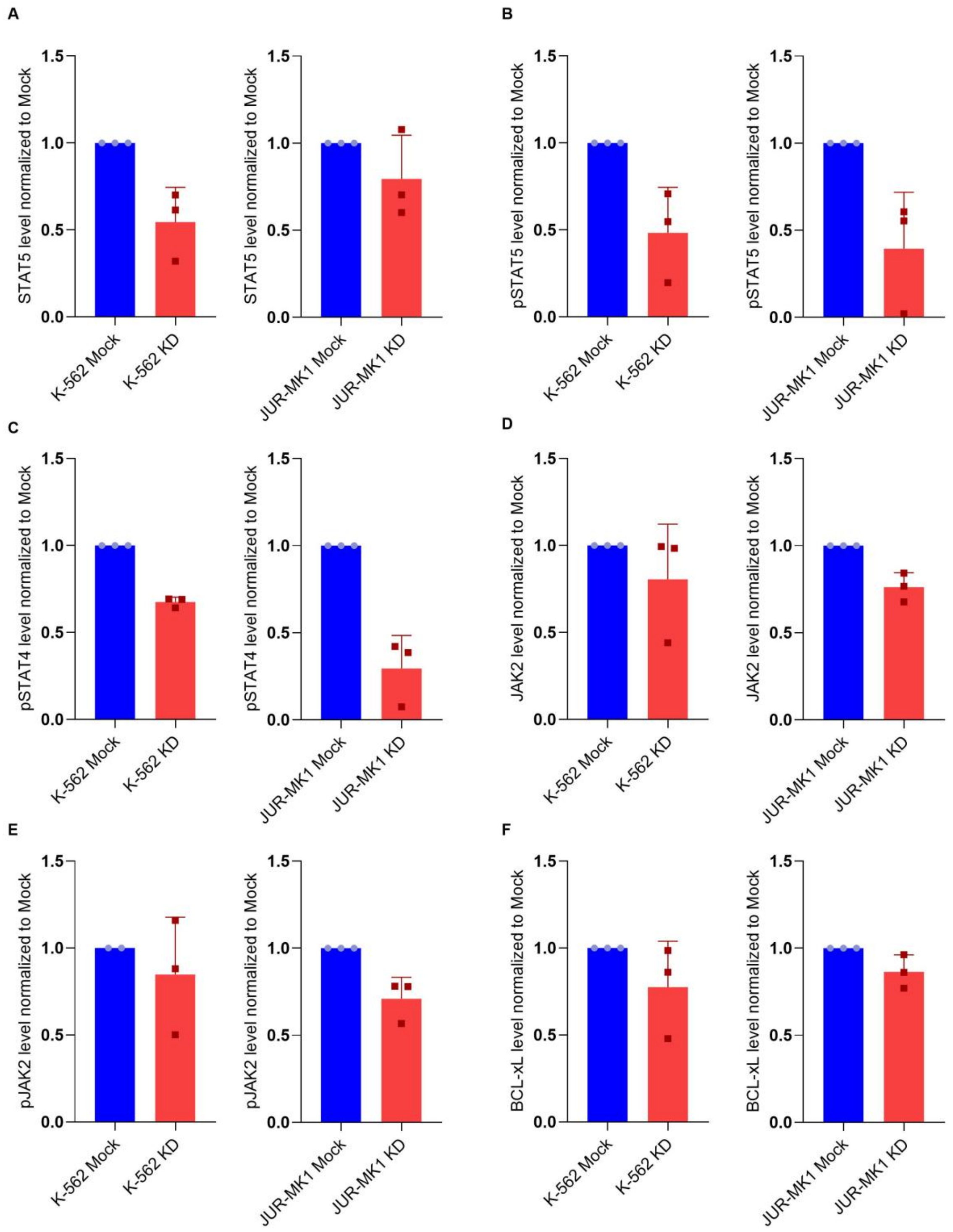

Figure S4. Quantification of JAK2-STAT5 pathway components following LEDGF/p75 depletion in CML cell lines. Densitometric quantification of immunoblot signals from Figure 5F in K-562 and JURL-MK1 cells transduced with control (Mock), knockdown (KD) vectors. Protein levels were normalized to the corresponding loading control and expressed relative to the Mock condition. (A) STAT5, (B) pSTAT5, (C) pSTAT4, (D) JAK2, (E) pJAK2, (F) BCL-

xL (BCL2L1). Bar represents mean ± SD of individual biological replicates (N=3).

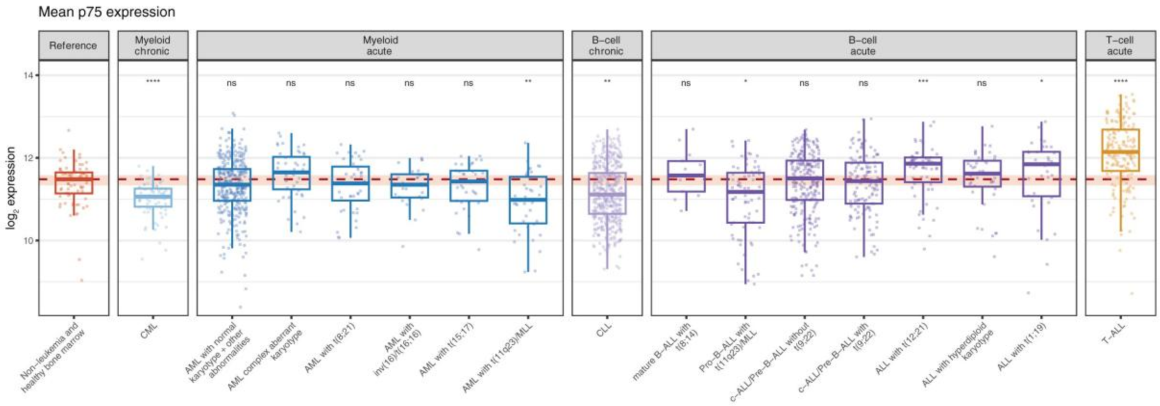

Figure S5. LEDGF/p75 expression in microarray across hematological malignancies in the MILE cohort (GSE13159) [29] . LEDGF/p75 expression was defined as the mean expression of two p75-specific PSIP1 probe sets (205961_s_at and 209337_at), which target the p75-specific 3′UTR. Dots represent individual patient samples; boxplots show the distribution of mean LEDGF/p75 expression per class (n = 1,890). The red dashed line and the shaded area indicate the median expression at its 95% bootstrap confidence interval of non-leukemic reference (n=74). Subtypes were compared with the non leukemic reference group using a two-sided Wilcoxon rank-sum test with Benjamini–Hochberg correction. Statistics ns, not significant; *$p < 0.05$, **$p < 0.01$, ***$p < 0.001$, ****$p < 0.0001$.
